# Efficiency of Health Care Risk Waste Management in Rural Healthcare Facilities of South Africa: An Assessment of Selected Facilities in Vhembe District, Limpopo Province

**DOI:** 10.3390/ijerph16122199

**Published:** 2019-06-21

**Authors:** Foluke C. Olaniyi, Jason S. Ogola, Takalani G. Tshitangano

**Affiliations:** 1 Department of Public Health, School of Health Sciences, University of Venda, Thohoyandou 0950, South Africa; Takalani.Tshitangano@univen.ac.za; 2 Department of Mining and Environmental Geology, School of Environmental Sciences, University of Venda, Thohoyandou 0950, South Africa; Jason.Ogola@univen.ac.za

**Keywords:** healthcare risk waste, open burning, cradle to grave, waste management

## Abstract

Waste generated form healthcare facilities is a potential source of health risks to the public, if it is not properly handled from the point of generation to disposal. This study was conducted to assess the efficiency of healthcare risk waste (HCRW) management in Vhembe District of Limpopo Province, South Africa. Fifteen healthcare facilities were selected in Vhembe District for this study. Data were obtained through in-depth interviews, semi-structured questionnaires, observation and pictures. Qualitative data were thematically analyzed, while the quantitative data were analyzed using the Statistical Package for the Social Sciences, version 25. In all the healthcare facilities; mismanagement of HCRW was noted at different points along the management chain. Poor segregation, overfilling of waste bins, inappropriate transportation and storage of waste in substandard storage rooms were observed in the facilities. All the waste from the district are transported to a private-owned treatment facility outside the district, where they are mainly incinerated. Enforcement of healthcare risk waste guidelines, provision of standardized equipment for temporary storage, empowerment of each healthcare facility to treat at least some of the waste, and employment of non-burn techniques for treatment of waste are recommended for more efficient management of healthcare risk waste in Vhembe District.

## 1. Introduction

Medical waste has been defined by the World Health Organization (WHO) as “all waste generated within healthcare facilities, research-centers, and laboratories related to medical procedures; including the same types of waste generated from other scattered sources and homes” [1]. A larger proportion (75–90%) of this waste, comparable to household waste and which can be managed along with other types of municipal waste are referred to as healthcare general waste, while the smaller percentage (10–15%), which constitute risks to the environment and human health are referred to as health care risk waste (HCRW) [2]. HCRW is also subdivided into various sub-categories based on the source, nature and effects of the waste: Infectious, sharps, pathological, chemical, radioactive, pharmaceutical, cytotoxic, and genotoxic waste [1].

**Sharps** waste include instruments that can cause cuts and puncture wounds. They include needles, scalpels, broken glasses, knives, etc.

**Vials** waste are bottle containers of injectable medications, which have been emptied. When they are intact, they are classified as hazardous waste because of their content. They are not disposed along with sharps unless they are broken. However, they are also not disposed with other infectious waste because of their potential to break and cause puncture injuries. They are separately disposed into puncture-proof containers.

**Infectious** waste comprises blood-stained materials, used syringes without needles, dialysis disposable equipment, materials containing excretions, waste from theatres and autopsies.

**Pathological** waste consists of human and animal tissues and organs e.g., placenta, amputated limbs, and resected internal organs.

**Pharmaceutical** waste includes expired, unused or contaminated pharmaceutical products also known as obsolete stock.

For the purpose of treatment, these wastes can also be sub-classified into: hazardous waste (waste which poses any form of risk to the waste generator, handler and the community, for example, pharmaceutical and chemical wastes) and biohazardous waste (waste which carries the risk of transmission of infections: infectious waste and sharps, which are contaminated with human tissue or body fluids). The mode and cost of treatment and disposal of hazardous and bio-hazardous waste are different.

Improper management of HCRW from the point of generation to disposal has been linked with health hazards to waste generators, handlers, and the community [3]. Healthcare workers are faced with the risk of being pricked by improperly disposed sharps waste and this exposes them to blood-borne infectious diseases notable among which are: Human Immunodeficiency Virus/Acquired Immune Deficiency Syndrome (HIV/AIDS) and Hepatitis. Needle prick injuries have been reported among both HCRW generators (nurses, doctors), handlers (cleaners) and scavengers who manually sort waste disposed on landfills from healthcare institutions [4,5].

A needle stick injury from an infected patient exposes a victim to 30% risk of contracting Hepatitis B virus and 0.3% risk of contracting HIV [5]. Other health risks include chemical burns, exposure to toxic pharmaceutical products and air pollution during treatment of such waste if burns methods like open burning or incineration are employed [5]. Furthermore, metals and toxic substances like dioxins which have been linked to cancer, immune system disorders, diabetes, and birth defects are released into the environment when HCRW are treated with incinerators which do not comply with standard emission standards [6]. With open dumping or dumping of waste in water bodies, contamination of drinking water can occur and expose the public to the risk of a wide range of infections. These risks can be minimized or completely eliminated if the waste is properly managed.

Management of HCRW involves a safe handling of the waste from the point of generation to the point of treatment and eventual disposal with minimal contact with the generators and handlers as well as the community. This involves minimization and segregation from source, safe transportation, temporary storage, offsite transportation, treatment and disposal. Each of these important stages have guidelines and standards controlling them which must be observed for the waste to be said to have been handled properly.

In South Africa, waste prevention, minimization and reuse were accorded a priority consideration on solid waste management [7]. This shows that the country regarded the reduction from source as a very important step in the management of any type of solid waste.

The Department of Environmental Affairs (DEA) of South Africa has been on the forefront of drafting waste (including HCRW) management guidelines in South Africa, as well as monitoring and conducting relevant studies on the subject nationwide [8]. The Department has drafted a policy document, which recognizes the Acts of South Africa which serve as the bedrock upon which the HCRW management policies are framed. The document also included guidelines on handling, storage, transportation and disposal of HCRW management as well as standards for the equipment to be employed in HCRW management. From this document, the Department of Health (DoH) of each province of South Africa has extracted their own guideline documents, with more details on the definition, handling, segregation, containerization, onsite transportation, and storage of HCRW within their healthcare facilities [9].

The Health Professional Council of South Africa has also developed a comprehensive healthcare risk waste management guideline booklet for healthcare professionals [10]. The guideline provided a full definition of HCRW and classified the waste into the various sub-categories. It also discusses the risks of mismanagement of the waste to the society and highlighted the roles and responsibilities of each health worker in proper HCRW management. Details on proper handling of the waste from the point of generation to final disposal can also be assessed in the booklet.

The continual rise in the quantity of HCRW being generated from healthcare facilities has been linked to consistent population growth, which results in an increase in the number of people who require the services of healthcare facilities and the encouragement of disposable medical equipment over reusable ones [8,11].

In 2008, the Department of Environmental Affairs and Tourism (DEAT) of South Africa reported that with an envisaged annual increase in human population by 1.06%, the rate of generation of HCRW in South Africa will also increase annually by 1.5%, using the 42,200 tons generated in 2007 as a template [8]. However, the quantity of waste generated from the country exceeded this projection because in 2014 and 2017; 44,139 tons and 48,749 tons, respectively were generated [12,13].

More demand for reusable equipment with refillable packaging, as opposed to disposable equipment has been reported in America and other parts of the world due to the availability of modern technologies for sterilization and disinfection [11]. This would greatly assist to achieve the source-reduction goal of HCRW management.

Efficient management of HCRW requires a lot of resources [14], thus, many developing, resource-constrained countries are reportedly facing many challenges of managing their HCRW [15]. Previous studies on HCRW management in different provinces of South Africa have mainly focused on hospitals [16,17,18,19] while smaller healthcare facilities like clinics and community health centers are rarely included in the studies. The same trend has been observed in Limpopo Province.

The Limpopo province of South Africa is a largely rural province with more than 87% of people in the province living in the rural areas [20]. The province is made up of five District Municipalities and 25 local municipalities. This study included the three basic categories of healthcare facilities (hospitals, clinics, and community health centers) in Vhembe District of Limpopo Province and it was conducted to assess the efficiency of HCRW management in Vhembe District in terms of compliance to HCRW management guidelines, HCRW segregation, onsite transportation, onsite temporary storage of HCRW, and the final disposal of HCRW generated from Vhembe District Healthcare facilities.

## 2. Methods

**Figure 1:**
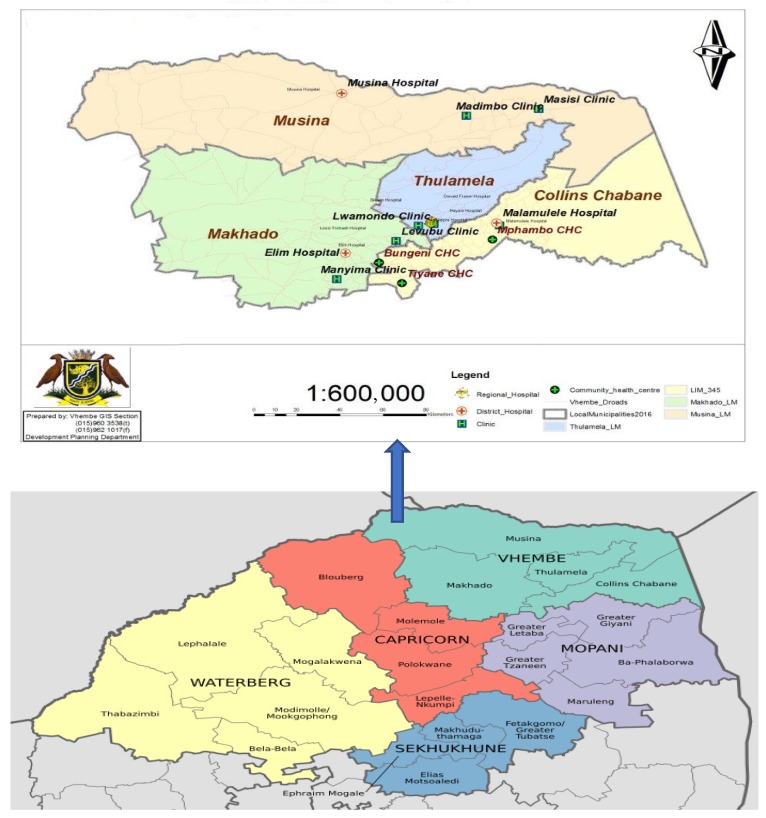
Map of Vhembe District in Limpopo Province showing the study areas.

A mixed method approach was employed to collect both qualitative and quantitative data from 15 healthcare facilities in Vhembe District Municipality of Limpopo Province, South Africa. Both types of study approaches were used to validate the results obtained. There have been many reports of poor management of HCRW from health institutions in Limpopo Province, as well as Vhembe District Municipality in the past [19,21,22], as well as undocumented reports of discovery of HCRW in some water bodies in the District. The District is made up of 4 local municipalities and 126 public healthcare facilities including District Hospitals, Clinics and Community Health Centers (Figure 1). There are also private owned surgeries, laboratories, pharmacy stores and other sources where HCRW are generated in small quantities, however, the public institutions were selected for ease of access.

A District hospital (DH), 2 clinics and 1 community health center (CHC) were randomly selected from each of the local municipalities for this study to ensure that the result can be generalized to the entire District. Thus, 4 District hospitals, 8 clinics and 3 CHCs were sampled, because one of the local municipalities had no community health centers. The population included all administrative heads of the selected facilities and HCRW generators and handlers in the facilities. A record obtained from the Department of Health section of the Vhembe District municipality just before data collection shows that there are 6074 health workers in the district, including the support staff (cleaners, community health workers, etc.). The Sloving’s formula

[*n* = *N*/(1 + *N*e^2^)] (1)

where *n* = sample size, *N* = total population and e = margin of error, set at 0.05 was used to calculate the sample size from this figure to obtain a sample size of 375, which was distributed over the different categories of HCRW generators and handlers (Table 1).

An interview guide was used to collect qualitative data from Infection Prevention and Control Practitioners (IPCCs) and Environmental Health Practitioners (EHPs) at the hospitals, as well as the managers of the clinics and community health centers. The interviews were conducted with consent and recorded on tape. For the purpose of anonymity, the participants were identified with codes, rather than names. The code is made up of the position of the participant and a unique number (The Managers were coded as Manager 1–7; IPPC 1–3 and EHP 1–4). Thematic analysis was employed for the qualitative data.

A total of 413 questionnaires were initially printed for distribution. This is because 38 questionnaires (10% of 375) were added to the original sample size to accommodate non-responses and invalid questionnaires, which lack vital information. However, many questionnaires were not returned; thus, the researcher ended up administering more than 500 semi-structured questionnaires to obtain only 229 which were well completed and fit for analysis. The initial plan was to administer the questionnaires and wait to collect them, however, most healthcare workers, especially doctors and nurses were unable to complete the questionnaires immediately because of their duties, thus, the researcher had to leave the questionnaires with them and return at a set date to pick them up. Many of the questionnaires were lost in this process and some were retrieved uncompleted, necessitating the administration of the same to another volunteered healthcare worker. The 229 completed questionnaires account for a response rate of 61.1%. Analysis was done using the Statistical Package for the Social Sciences (SPSS), version 25. A digital camera and an observation checklist were handy throughout the field work to record all observations and take relevant pictures in order to validate or reject the responses obtained during the interviews and through the questionnaires.

Ethical clearance and permission to conduct the study were obtained from University of Venda Research Ethics Committee, Limpopo Provincial Department of Health, Vhembe District Executive Manager, Chief Executive Officers of all the hospitals and the Managers of Clinics and Community Health Centers before data collection was commenced.

## 3. Results

In Limpopo Province, the DoH developed a guideline for HCRW management and made the guideline available at all healthcare facilities in the province. The Department also contracted a waste management company for transportation of HCRW out of every public healthcare facility, treatment and disposal of the waste. The Department is responsible for the payment of all the equipment supplied by the waste management company, treatment cost of HCRW as well as Personal Protective Equipment (PPE) and immunization for health workers.

### 3.1. Demographic Characteristics of Respondents

A total of 229 healthcare workers (150 professional nurses, 11 student nurses, 28 doctors, 28 cleaners, 10 community health workers and two pharmacy staff) participated in this study. The population was dominated by females (*n* = 194: 84.7%) and their ages range between 18 and 64 years (mean = 40.0). With the exception of the student nurses who had only been at the hospitals for two weeks, more than half (*n* = 132, 65.67%) of the other respondents had 1–10 years of professional experience; while 39 (19.40%) had 11–20 years and 30 (14.93%) had 21–30 years professional experience in their respective healthcare facilities.

### 3.2. Healthcare Risk Waste Generation

The rate of generation of HCRW in healthcare facilities of Vhembe District of Limpopo Province is dependent on the type (or hierarchy) of healthcare facility, the type of services being rendered, the number of patients attended to on a daily basis and whether or not there is provision for admission of patients. In the literature, the quantity of HCRW generated is usually calculated in terms of “daily HCRW mass per bed per day” [14]. However, the rate of generation of HCRW in this study was calculated based on “mass per patient per day” to be able to accommodate the healthcare facilities sampled in this study, which do not have “beds” as they do not admit patients.

In all the healthcare facilities in Vhembe District, the local municipal governments are responsible for the disposal of healthcare general waste, which they pick up from each facility at scheduled days of the week, when they pick up domestic waste from other houses within the local municipalities. However, a waste management company has been contracted to pick up HCRW from all the public healthcare facilities in Limpopo Province for subsequent treatment and disposal.

Measurement of the quantity of HCRW generated is not done directly at the facility level, but the measurement is taken by the representative of the waste management company in the presence of a representative of the healthcare facility, usually the Environmental Health Practitioners in the hospitals and any delegated nurse at clinics and CHCs. A copy of the record of the quantity of the HCRW collected is then left at the healthcare facility. These records are usually kept in a waste management folder. These records were then assessed to calculate the average quantity of HCRW generated in each of the sampled facilities.

The regularity of weighing is directly proportional to the frequency of visit of the waste management company to the healthcare facility. The company has different schedules for the healthcare facilities based on the quantity of HCRW they generate: in the District hospitals, the waste is weighed twice or three times a week, while at the clinics and CHCs, the schedule is either weekly, fortnightly or, rarely, monthly. However, these disparities were adjusted while calculating the quantity of waste generated based on the figures obtained at each facility at different times, to have a uniform ground for all the facilities. The calculation was finalized on a daily basis.

In the district hospitals, which provide all forms of medical treatments including surgeries—more than 600 patients are attended to every day (this number includes patients on admission and outpatients), an average of 338.15 kg of HCRW is generated every day. This accounts for 0.54/kg per patient per day.

The services provided by the clinics include treatment of common and chronic illnesses such as common cold, sexually transmitted infections, high blood pressure, diabetes, as well as some acute disorders and antenatal care services. Some of them also run Youth friendly clinics, HIV Voluntary Counselling and Treatment services. While some of the clinics conduct delivery services for women in labor, others close to hospitals do not conduct deliveries, but refer their patients in labor to the nearest hospital. The clinics do not provide in-patient admission services except for post-natal women, who are admitted for observation after delivery and discharged after being certified stable within a few hours of delivery. With an estimated number of 83–155 patients and clients (clients are those who visit the hospital for counselling and testing purposes, which may not require any form of treatment) visiting a clinic per day, each clinic generates an average of 15.5 kg of waste daily.

The services provided by the CHCs are similar to those obtainable at the clinics. However, these facilities tend to serve more people in the community than the clinics as they attend to as many as 350–400 patients and clients per day. They also do not also provide in-patient admission services, except for post-natal women who are delivered within their facilities. Each of these facilities generate an average of 19.2 kg per day.

Table 2 shows the average number of patients being seen at the healthcare facilities on a daily basis with their HCRW generation capacities.

Pharmaceutical waste is not disposed directly by hospitals, clinics and CHCs in Vhembe District, rather, the waste is transported to the Chief Pharmacist at the Regional Hospital who usually apply for an approval to destroy it from the Provincial Government and can only release it to the waste management company for destruction after obtaining such approval.

Healthcare staff in Vhembe District do not understand how the terms “minimization and reduction of waste” apply to medical waste. Participants were surprised to hear about the issue of reduction of HCRW. When an Environmental Health Practitioner (EHP) was asked how they achieve waste minimization in her healthcare facility, she laughed when she responded:

*“No, we don’t do those here. It’s not easy to minimize waste, is it not medical waste? It’s not easy. People are getting sick every day, that’s why we cannot. We can’t minimize diseases, no, no, no. You need to see how many people are being admitted into the hospitals”*

(EHP 2)

An IPCC had the same disposition.

*“Reduce waste?... that would not be in our capacity. I mean, we are following the guideline. The guideline says, “take a bottle, put it in the container, we will come and collect”, that’s all”*

(IPCC 1)

### 3.3. Healthcare Risk Waste Segregation and Filling of Temporary Storage Bins

The equipment supplied to Vhembe District healthcare facilities for temporary storage of HCRW include labelled color-coded bins (yellow bins with red liners for infectious waste, labelled yellow bins for sharps and vials waste and green bins for pharmaceutical waste). These are to assist the staff to effectively segregate medical waste from source, such that general waste and the various sub-categories of HCRW are dropped into separate containers at the point of generation. The EHPs in the hospitals and the managers at clinics and CHCs acquire these equipment from the service provider, store them temporarily in their offices or stores and distribute to the various sites where they are needed in their healthcare facilities. In the hospitals, IPCCs often assist or oversee the activities of the EHPs in this regard. These staff are also responsible for record keeping of the receipt and use of the equipment.

Good segregation practices depend on the knowledge of an HCRW waste generator about the various subcategories of HCRW. Almost all the respondents (*n* = 204, 92.3%) indicated that they generate both HCGW and HCRW in their facilities. However, on the question on identification of the various subcategories of HCRW, the nurses and cleaners excelled in the identification, while most of the doctors could not identify all the subcategories. There is a statistically significant difference in the level of knowledge of nurses and cleaners about HCRW categories as compared with doctors (*p* = 0.000).

All the respondents claim that they segregate medical waste from general waste and they also segregate HCRW into their different sub-categories from the point of generation. However, when asked to rate HCRW segregation practices in their facilities, 65 (28.4%) rated their facilities “excellent”, while a larger percentage rated them “good” (*n* = 73, 31.9%) or “very good” (*n* = 72, 31.4%). Only 4 (1.7%) respondents rated their facility “poor”. Some of the respondents who rated their facilities “poor” were engaged in verbal discussions on the reason why they rate their facility so, and the common response was:

*“We are trying, but, we are not perfect. Sometimes, we forget”*

(Respondents to questionnaires)

The Clinic Managers, EHPs and IPCCs were asked if healthcare staff in their facilities always comply to the HCRW guidelines in terms of waste segregation and filling of bins, they all ascertained that they frequently witness poor segregation of general waste from HCRW, as well as mixing different categories of HCRW. However, some nurses blame the mixing on patients, doctors and student nurses. Filling of the temporary storage bins beyond the recommended and demarcated ¾ level have also been noticed. Below are extracts from the interviews conducted for them:

*“Yes, we do comply, but sometimes we do find mistakes of mixing general waste and medical waste and also mixing of other sub-categories of medical waste”*

(Clinic Manager 1)

*“Mixing of vials and sharps—sometimes, you find that they are not segregating the waste properly. Another thing is that we find that the waste bins are full beyond the limits that we are expected not to exceed”*

(CHC Manager 1)

*“…the thing is, at the Paediatric’s ward, they are admitting children and their mothers. The mothers are not trained on how to segregate the waste, so the mothers are the ones mixing the waste…I don’t know how we can train them…maybe staff members can give them a lecture of 5 min to mothers each and every morning, because today, it’s one mother, next day, it’s another one, so, you have to do it every day, for it to work. In other wards, they do comply”*

(EHP 4)

*“They mix general and medical waste. Sometimes, sharps with medical (infectious) waste. As you can see in our office, we are having different kinds of bins, each of them has its own designation. Each of them has also been assigned to different wards…but it happens that when you check the waste, you will find the bandages in the sharps container, where they are not supposed to be, sometimes, they complained that it is the doctors or the student nurses…and most of the time, it’s just the staff’s attitude. It’s not something that you can say you can’t do. It’s not difficult”*

(IPCC 2)

With further probing on the attitudes of healthcare workers to training on healthcare risk waste management, doctors were identified as a group of profession who do not attend trainings on HCRW management whenever a call was made for such, because they usually claim that they are very busy.

*“Every time, they say ‘we are busy’. Okay, the service provider will say, “okay, tell us, make an appointment, we can come any day, specifically for you doctors”, but the reply will be “Ah, there is this infection control nurse. She is going to tell us everything”, but, every time, they are busy, you won’t get them”*

(IPCC 2)

A doctor in one hospital confirmed her statement when he was given a questionnaire to complete. He said:

*“These questions are not relevant to doctors. You should ask the nurses and cleaners. As for me, I have nothing to do with waste. Each morning, I come to find an empty waste bin at my work station, see my patients, drop the waste and leave. The next morning, I find the bin empty again, whatever happened to the waste is not part of my job description”*

(A doctor)

**Figure 2:**
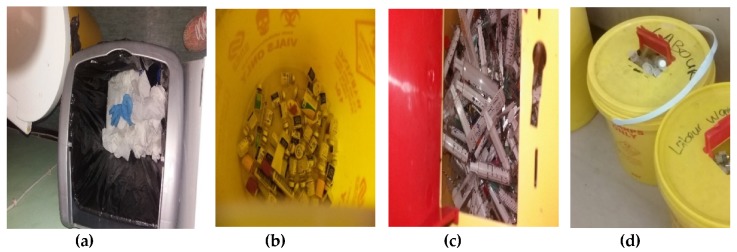
Photos taken by researcher during field work showing: healthcare risk waste (HCRW) used gloves mixed with general waste in a general waste bin (**a**); mixing of infectious waste (soiled cotton wool) with sharps (glass tube) and vials waste (**b**); mixing of general waste (paper package of syringe) with sharps (needles) (**c**) and filling of HCRW bin beyond the ¾ recommended level (**d**).

Some pictures taken onsite confirmed the mixing of general and HCRW waste, mixing of the various subcategories of HCRW and inappropriate filling of the HCRW bins (Figure 2).

### 3.4. Temporary Storage

Sharps waste are temporarily stored in puncture-proof containers, while other types of HCRW are separately stored in color-coded bins at the point of generation. In some of the facilities, some staff were observed to be without personal protective equipment and some categories of HCRW were not dumped into the appropriate bins. Some of the temporary storage bins were not properly closed and yet they were being put to use.

**Figure 3:**
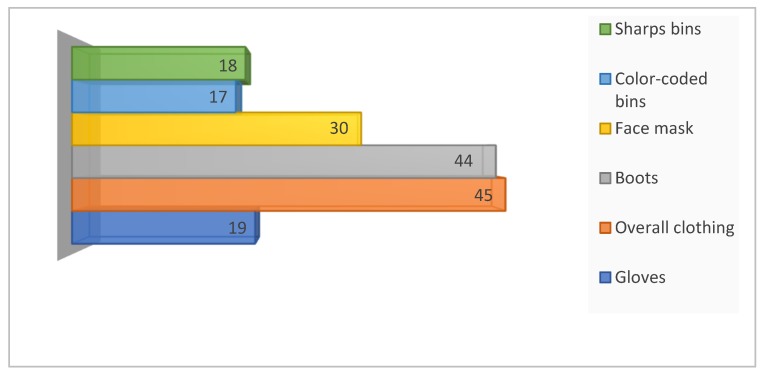
Number of healthcare staff who reported insufficient equipment for HCRW segregation and transportation and personal protection.

Shortage of personal protective equipment and HCRW management equipment were reported by some staff in the clinics and community health centers (Figure 3).

However, all the EHPs and IPCCs reported sufficiency with temporary storage equipment in the hospitals.

*“I make requisition for the bins from the service provider, but, it is the cleaners who distribute them to the wards wherever they are needed. So, we don’t have shortage”*

(EHP 2)

*“Yes. I don’t have problems with the bins”*

(IPCC 1)

*“We have enough bins”*

(IPCC 3)

While some participants reported malfunction of some of the bins—lids not closing properly and broken pedals, the EHPs and IPCCs ascertained that there is nothing wrong with the lids, only that the staff are ignorant on how to close them.

*“Sometimes, you find that the lids are not fitting properly”*

(CHC Manager 1)

*“… you can find that the containers or bins for collecting waste are not functioning well, like when you are not supposed to use your hand to open the bin, but you find that the pedal is not working”*

(Clinic Manager 2)

*“I think it’s the personnel who do not know how to close the lids properly. I always help them and show them how to close it. I was once called at the Maternity ward because they could not close the lid. The problem is not with the equipment.”*

(EHP 1)

*“The only problem, perhaps, is that the staff need training every now and then on how to use the bins, some of them are not competent, so they break the pedals. When the company comes, they stress much to demonstrate…but, it’s improving now”*

(IPCC 1)

In some facilities, the equipment was being put to other use, like fetching water for washing cars or as containers of water in the bathrooms. An EHP lamented about this when she was asked “What are the challenges you have been facing while discharging your duties on HCRW management?”:

*“My major challenge is with the medical waste equipment, what I found is that, they do not use them for specific purposes. If I say, these are for sharps, sometimes, you find people using them to wash cars, sometimes, they are using them maybe to store their polishes, I have been trained to write out those ones that are outdated, old ones and all that. But, come again two weeks’ time, you will still find out they are using them for the wrong things. I get tired of talking to them, but I am trying. Sometimes, they order more bins than they need, keep them in their store and use them for other purposes. Even if I close them, they cut out the lids. They cut, so that they can open it, I almost fainted …”*

(EHP 2)

**Figure 4:**
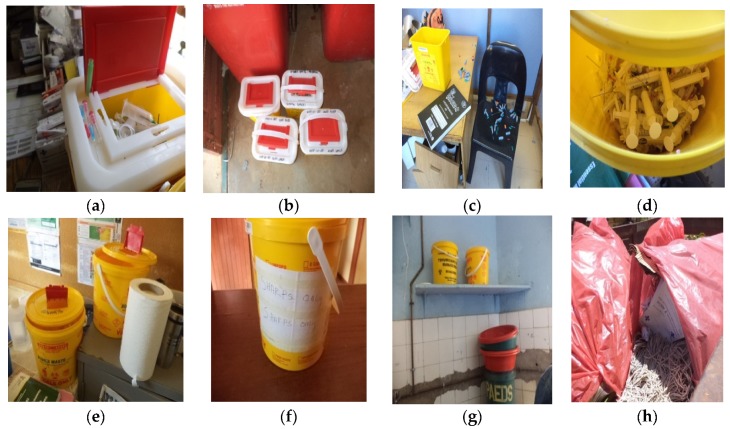
Photos taken by researcher during field work showing: careless dropping of sharps waste on top of the waste bin (**a**); improper closure of the lids of containers for sharps waste (**b**–**e**); accidental spillage of sharps waste due to improper closure of the lid (**c**); a bin for temporary storage of vials waste, relabeled to be used for storage of sharps (**f**); temporary storage equipment being used as water containers in the bathroom (**g**) and inappropriate use of red liners for storage of general waste (**h**).

All these were confirmed during field work (Figure 4).

### 3.5. Onsite Transportation

**Figure 5:**
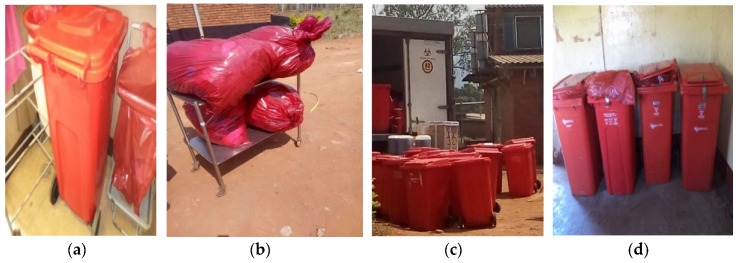
Photos taken by researcher during field work showing onsite transportation of HCRW: Wheelie bin in the sluice room of a healthcare facility (**a**); medication trolley being used to transport HCRW to the central storage area (**b**); HCRW being transported out of healthcare facility with wheelie bins (**c**) and overfull wheelie bins and some with faulty locks (**d**).

Transportation of HCRW from the point of generation to the central storage areas of healthcare facilities in Vhembe District is a duty of cleaners and ward aides. In some very remote clinics, volunteers from the community assist to carry out these duties. Majority of the respondents (*n* = 164, 71.6%) stated that they make use of dedicated trolleys (large wheelie bins: Figure 5a) for this purpose, while 28 (12.2%) and 22 (9.6%) respondents respectively indicated that they use any available container for the transportation or the waste is being manually carried to the central storage room by the cleaners. In a facility, a trolley which was being used for dressing of wounds was observed also being used to transport HCRW to the central storage room (Figure 5b).

Infectious waste is transported to the HCRW treatment facility in the wheelie bins while new bins are supplied to the healthcare facilities (Figure 5c). The wheelie bins are equipped with locks to ensure that they can be locked after infectious HCRW has been stored in them. To the question, “how do you transport your waste from the point of generation to the central storage area?”, an IPCC responded:

*“The service provider has provided us with the bins. They call it the wheelie bins—the red, big ones. Every ward is having the bin and they keep them in the sluice rooms. After dropping medical waste there, they wheel it from the wards into the central storage area somewhere there (pointing in the direction of the site of central storage area)”*

(IPCC 3)

However, some of the wheelie bins were found to have broken locks while some were too full to be lockable (Figure 5d).

### 3.6. Central Storage

All the HCRW generated in every section of the healthcare facilities are transported to a central storage site within the facility from where the waste management company picks them up. In the district hospitals, these sites are small buildings with sufficient space dedicated for this purpose alone. However, in the clinics and CHCs, the storage rooms are usually old toilets or old incinerator rooms converted to storage areas or a simple fence without a roof, but with a door or an iron gate. In some clinics, the same room is being used for the storage of gardening and cleaning materials. In all the facilities, there are danger signs at the entrance of the rooms or posted on the doors to keep unauthorized persons away. These signs were provided by the waste management company on contract with Vhembe District.

More than half of the respondents (*n* = 172, 75.1%) claimed that they were aware that they have central storage areas in their facilities and the storage areas are not accessible to the public. In one hospital, the researcher and her assistant were not allowed to move close to the storage room “for safety purposes”. We were told that access to the room can only be granted if we were properly dressed in protective gowns with gloves, boots and face mask. But, such was not the case in the clinics and CHCs where some of the central storage areas were found to be without functioning doors, keys or roofs or with broken windows which make access to them easy.

To the question: “Is your central storage room secure and up to the recommended standard?”, some managers responded:

*“Yes, we do have a particular site which is always locked. We gave our cleaners the responsibility to collect those full buckets, so, it means that the professional nurse or who is responsible for the consultation will close that lid and from there the cleaner will collect the bucket and take it to where it is supposed to be stored until the service provider collects it”*

(CHC Manager 1)

*“...we also don’t have good storage areas, we improvised, we just found a room to store the waste, not even having a locker”*

(Clinic Manager 2)

*“The door is there but it does not have a locker, it’s not really up to the standard. We are just happy that we have a storage, because at first, we were using one of the toilets, we are just happy because it’s not tissue waste, because the refrigerator is inside. But because the storage room is not locked any person can come in, which is not safe for the community as well”*

(Clinic Manager 3)

A manager of a CHC expressed her concern over the lack of a standard central storage room in their healthcare facility:

*“We have a case when a man from the community sneaked into our central storage room because the lock is bad. He opened the buckets where we kept the sharps and vials, poured the needles and vials on the floor and ran away with the buckets. You can imagine the risks we are exposed to when we tried to put those things in other bins”*

(CHC Manager 2)

**Figure 6:**
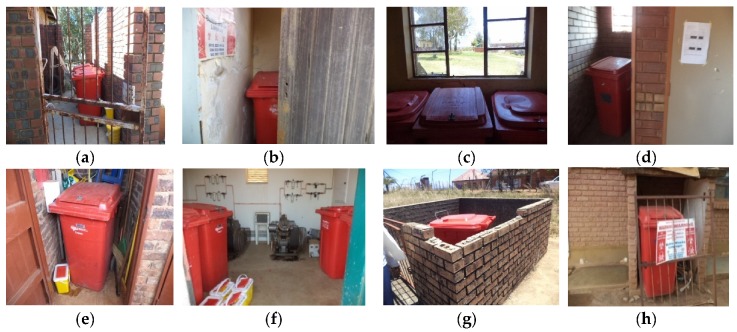
Photos taken by researcher during field work showing central storage areas for HCRW in Vhembe District Healthcare Facilities: HCRW storage rooms with doors, but, without locks (**a**,**b**); HCRW storage room without a door (**c**); HCRW stored in the same room with cleaning and gardening equipment (**d**); HCRW storage room with a broken window (**e**); an old incinerator room converted to storage area (**f**); HCRW storage area with a gate, a low fence but, without a roof (**g**); HCRW being stored by the corner of a wall, not in a secure room (**h**).

Figure 6 shows the conditions of some central storage areas.

### 3.7. Offsite Transportation

HCRW generated in healthcare facilities in Vhembe District are transported out of the facilities by the waste company on contract with the district. This company has an agreement with each facility about how frequently they need to collect waste, based on the size of the facility and the quantity of waste they generate. Thus, waste is being collected from the district hospitals two or three times a week and from the clinics and CHCs, weekly, fortnightly or monthly. The schedule is constant in the hospitals where there are specific days of the week when the waste is picked up in the morning. When the Environmental Health Practitioners were asked if waste company always adhere to their scheduled time of picking the waste, they responded:

*“Always. They never fail. Even if the days fall on public holidays, we will come to find that they have collected the waste”*

(EHP 2)

*“No. They don’t fail. At all, at all. Even during holidays, they do come”*

(EHP 3)

However, in the clinics and CHCs, the schedule is not as constant. Some managers claim that they are only sure the company will come once in two weeks, but they are not sure of any particular day they would come. Others claim the service providers are regular, visiting weekly, fortnightly or once in three weeks. Few respondents (*n* = 43, 18.8%) claim that their waste was not being removed offsite regularly, although none of the facilities reported ever having an overload of HCRW. A manager was asked: “Do you sometimes have an overload of HCRW in the central storage room before they are picked up?”, she responded in the negative.

*“No, even though they come fortnightly, when we have a load of medical waste, we do call them even though it is not due time for them. They indicated that ‘you can even call us if you have an overload of medical waste”*

(CHC Manager 1)

### 3.8. Treatment and Disposal

The waste management company has been saddled with the responsibility of treating and disposing all HCRW generated in the District. Each local municipality is also expected to dispose the general waste generated from the facilities like other household waste. A total of 180 (78.6%) respondents claim that they do not dispose any waste at all in their facilities because the Municipality officers pick up the general waste for disposal while the service provider takes care of the HCRW. However, 32 (14%) affirmed that they dispose some of the waste in their facilities by burning (7%) or incineration (6.6%). Few staff (*n* = 17, 7.4%) are unsure whether any type of waste is being disposed in their facilities.

**Figure 7:**
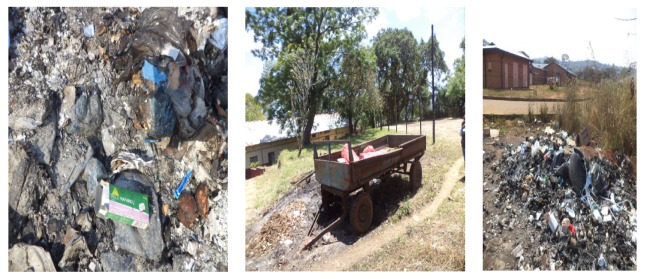
Burning sites within Vhembe healthcare facilities. (Photos taken during field work).

In some clinics and CHCs, small areas were observed within the compounds where some waste were being burnt (Figure 7).

### 3.9. Record Keeping

The EHP in the hospitals and managers in the clinics and CHCs are responsible for the keeping of the records of the quantity of HCRW generated and turned over to the waste company. The record is a form completed by the representative of the waste company, which shows the quantity of each category of HCRW present at the central storage room whenever the waste is being taken away for treatment and disposal. This form is completed each time the waste management company visits the healthcare facility and a copy is left with the EHP or the manager. At the end of every month, EHPs make a compilation of all the records to derive the monthly figure of HCRW generation in the hospital. This is not done at the clinics and CHCs.

The waste management company also sends a copy of the certificate of destruction to the District office on a monthly basis. The certificate contains information on how much of HCRW was transported out of Limpopo Province and the mode of treatment. EHPs, IPCCs and managers have access to these certificates through the District office. However, one of the EHPs and some managers are not aware of the existence of such a certificate and they do not have copies in their files. Below are extracts from the interviews when the participants were asked if they usually receive certificate of destruction from the waste management company.

*“Yes. I also have that one. I get it from the District. The service provider sends the certificate of destruction to the District and the District forward it to me”*

(EHP1)

*“Yes, they do. They are with the EHP and I also have my copies. They send the certificate to e-mails”*

(IPCC 1)

*“They email me the certificate”*

(Clinic Manager 2)

*“No. We only have the service level agreement that they will be working with us. They don’t provide us with anything that shows that we did this with the medical waste”*

(CHC Manager 1)

*“No. They don’t. I heard that there is a certificate, I have never seen it but I have heard about it. I think the certificate is via the District and I don’t know how regularly they give them”*

(Clinic Manager 3)

When the question was posed to an EHP, she shook her head in disbelief/confusion. After the researcher explained to her the content of the certificate and why it should be obtained, she asked:

*“Should that be every month or every year?” …what an interesting question! I will make a follow-up on that…or, maybe the infection control has that certificate”*

(EHP2)

### 3.10. Budget for Healthcare Risk Waste Management

The Provincial Department of Health is responsible for the acquisition of HCRW management equipment, as well as payment for the cost of treatment and disposal of HCRW. The healthcare facilities are not directly involved. The IPCCs and EHPs attested to this.

*“The Provincial Government. We order from the service provider, they deliver and then take the invoice to the provincial office”*

(IPCC 3)

*“The Department of Health in the Province. When I make the request, the service provider gives me a list of what they supply which I submit to the Department”*

(EHP 1)

## 4. Discussion

This study included more nurses as participants than healthcare staff from other professions. This is because nurses are usually more in number in healthcare facilities; they actually make up 72.5% of all the health workers on the register of Vhembe District Municipality [23].

Infectious waste was discovered as the major type of HCRW being generated at healthcare facilities in Vhembe District, as confirmed by other similar, previous studies [21,24]. This is understandable because most of the types of HCRW produced at healthcare facilities fall under this category and some of them are bulky, for example, diapers and other materials contaminated by excreta, linings from maternity wards contaminated with blood. In the hospitals, pathological waste is the next most bulky subcategory of HCRW generated, while this is not the case in clinics and health centers where only few deliveries are conducted and surgeries are not carried out.

Minimization of HCRW is not being practiced in Vhembe District. An IPCC claimed that they were simply following the guidelines. A scrutiny of the Provincial guideline confirms that there were no detailed instructions on how to reduce HCRW, though the document clearly states that: “Health care risk waste to be minimized and separated effectively in such a manner that the environment is not polluted” [25]. Also, the training manual provided by the waste management company does not address the issue of HCRW minimization. This means that this important step of HCRW management has been relegated to the background and would not be discussed whenever there is a training about HCRW management at the healthcare facilities. HCRW minimization has been proven to be effective in the reduction of the cost of HCRW treatment by up to R20,000 per month [4]

This study confirmed that mixing general waste and HCRW, as well as mixing of various sub-categories of HCRW occurs in healthcare facilities in Vhembe District. Some healthcare staff are unable to identify all the various categories of HCRW. Identification of the different subcategories of HCRW and segregating them into various appropriate containers from source is the key towards achieving the exact quantity of HCRW, which reach the treatment plant. Once HCRW has been mixed at source, it must remain mixed until it reaches the treatment facility because sorting of the waste into different categories after it has been mixed exposes whoever attempts to sort it to the risk of injuries from sharps or contact with hazardous chemicals. Mixing general waste with HCRW has been identified as a reason for a high cost of treating HCRW because once mixed, the entire waste stream has to be treated as hazardous [4,26].

Poor segregation practices among healthcare workers noted in this study has also been variously reported in many developing countries and linked to ignorance of the risks and costs associated with such practice and apathy towards the issue of HCRW management [9,27]. This increases the cost of treatment and disposal of such waste. Moreover, mixing different categories of HCRW, for example, mixing infectious waste (like bandages) with hazardous waste (like medication vials) requires that the mixed waste has to be first disinfected before being treated as hazardous waste. This amounts to a high cost of treatment, as it has been reported that it costs about three times more to treat infectious waste compared with other forms of hazardous waste from healthcare facilities [6].

This study identifies doctors as being the main category of healthcare profession more culpable in the mixing of HCRW, like other previous, similar studies [9,27,28] and uncovers the reason for this finding, as the attitude of doctors towards training on HCRW management: many doctors do not accept a responsibility of waste management as a part of their job description in their capacity as HCRW generators [19], therefore, they do not place any priority on attending trainings on HCRW management. Meanwhile the HPCSA recommends continual training for healthcare workers to keep themselves up to date with the latest scientific knowledge on the management of HCRW [10]. The Department of Health must make more deliberate efforts to train doctors, because training has proven fruitful in helping to improve doctors’ attitudes and practices towards HCRW management [29,30]. The use of attendance of HCRW management training programs as a way of generating continuing professional development (CPD) points for doctors could be an incentive to encourage them to attend the trainings [31].

It is noteworthy that in Vhembe District healthcare facilities, cleaners and volunteers are the ones assigned with the responsibility of transporting HCRW from the point of generation to the central storage area, according to this study. In some facilities, nurses were reported to be transporting the waste and this makes them susceptible to carrying infectious organisms back to the patients they take care of in the wards [4]. However, the fact that some waste handlers have to manually transport the waste from the point of generation to central storage rooms in remote healthcare facilities is a point of concern because of the risk of injuries to their legs and feet in cases of spill, or puncture wounds if the waste contain sharps, like needles, especially when they are not well protected by safety boots.

Moat central storage areas visited during the field work for this study do not meet up with the recommendations for a standard central storage room of a healthcare facility, which include among others: easy access by HCRW handlers and the service providers, sufficient space for storage of HCRW until transportation outside the facility, secured lock to prevent unauthorized access, appropriate ventilation and lighting [24]. This is because there was no plan for such structures while building the facilities. In most cases, old public toilets or store rooms for gardening are being improvised, as HCRW central storage rooms. In cases where there were no secure locks, relatively free access to the rooms poses a risk to members of the community who may not have knowledge about the risks of HCRW.

All HCRW generated from healthcare facilities in Vhembe District are being transported out of the District to a waste treatment center located in Capricorn District, another District Municipality in Limpopo Province. No part of the waste is being treated onsite. This practice has been reported to increase the cost of treatment of HCRW, which could be minimized if at least some of the waste is treated onsite [32]. Efficient onsite treatment modalities which do not require special training like shredding of sharps, disinfection and microwave can be employed in the healthcare facilities within the District to reduce the quantity of HCRW that is transported to the waste treatment facility. This would greatly reduce the cost of transportation, as well as allow the treatment facility to easily cope with the reduced quantity of waste it has to treat. However, the Department of Health must be willing to provide the initial cost of installation of these treatment modalities in the healthcare facilities.

According to the reports on the treatment certificates obtained from the waste management company, disinfection and incineration are the only methods being employed for treatment of HCRW transported out of the healthcare facilities in the District. Non-burn techniques are now being encouraged for the treatment of HCRW, to avoid the negative consequences of incineration, which include air pollution and release of toxic substances to the environment [6]. These techniques include:

- Low-heat thermal processes which involves the use of thermal energy at temperature range between 100 °C and 180 °C in moist or dry environment to destroy pathogens. This temperature is high enough to destroy most micro-organisms and yet not enough to cause combustion. Autoclaves and microwaves operate using this technique [6,33].
- Chemical processes where chemicals and disinfectants like sodium hypochlorite, sodium dioxide, peracetic acid or lime solution are react with the waste to destroy the constituent pathogens [6,34].
- Biological processes: enzyme mixtures are used to decompose organic matters [4,6].
- Irradiative processes: ionizing radiation and ultraviolet sources are used to destroy the micro-organisms e.g., electron beam radiation technology. However, this is an expensive technique [4,6].
- Mechanical processes which involve reducing the volume of the waste or rendering the waste unrecognizable, e.g., grinding, shredding, mixing, agitation, etc. [6]. However, after using this type of technique, another treatment method must be applied to render the waste non-hazardous.

Some of these non-burn techniques have been adopted in some provinces of South Africa including Gauteng and North West [12].

Some types of healthcare general waste, mostly boxes for packaging of medications were being burnt in some clinics and CHCs. The managers complained that the boxes were too big to be packed into local municipal waste carriage vehicles, hence the need to get rid of them at the healthcare facilities. However, the practice of open burning causes air pollution, which renders the environment unsafe. This practice is not acceptable because it violates the constitution of South Africa, which states that every South African has a right to a safe environment, which is not harmful [35].

The “polluter pays principle” of HCRW management states that “the waste generator must accept the complete financial culpability for the responsible handling storage, transportation, treatment and disposal of waste” [36]. Since the Provincial Department of Health is responsible for the payment of all the cost incurred in the management of medical waste generated by all public healthcare facilities in the Province, the Department must monitor all the activities of the waste management company on contract with them, to ensure that all the waste generated in the province is properly treated and safely disposed, to prevent further incidences of illegal dumping of untreated healthcare risk waste in the province.

## 5. Conclusions

This study has shown that HCRW is not being efficiently managed in Vhembe District of Limpopo Province, South Africa. It is recommended that the guidelines for management of HCRW are enforced and compliance ensured at all levels and among all the staff. Efforts at training of HCRW generators, with special attention on doctors should be intensified. Some form of treatment of HCRW should be permitted and encouraged in each healthcare facility and non-burn techniques of management of HCRW should be embraced in Limpopo Province.

## Figures and Tables

**Table 1:** The sampling frame.

Category of Health Worker	Population	Sample
Nurses	4403	252
Support staff	1481	81
Doctors	159	40
Pharmacy staff	31	2
Total	6074	375

**Table 2:** Healthcare risk waste (HCRW) generation figures in Vhembe District.

Rank of Facility	Average Number of Patients per Day	Types of HCRW Generated/kg/day				
		Sharps	Vials	Infectious	Pathological	Total
DH	631	13.75	12.4	277.0	35.0	338.15
Clinic	119	3.7	2.8	8.3	0.7	15.5
CHC	375	6.0	3.4	6.0	3.8	19.2

DH: District Hospital; CHC: Community Health Center.

## References

[1] Chartier Y., Emmanuel J., Pieper U., Pruss A., Rushbrook P., Stringer R., Townend W., Wilburn S., Zghondi R. (2014). Safe Management of Waste from Healthcare Activities.

[2] Yawson P. (2014). Assessment of Solid Waste Management in Healthcare Facilities in the Offinso Municipality. Master’s Thesis.

[3] Hangulu L., Akinola O. (2017). Health care waste management in community-based care: Experiences of community health workers in low resource communities in South Africa. BMC Public Health.

[4] Vumase B.S. (2009). An Evaluation of Operational and Administrative Procedures for Health Care Waste Management in Public District Hospitals of South Africa. Ph.D. Thesis.

[5] World Health Organization Fact Sheet (Health-Care Waste) 2018. https://www.who.int/news-room/fact-sheets/detail/health-care-waste.

[6] Jorge E., Hrdinka C., Ryder R., McKeon M., Berkemaier R. (2004). Non-Incineration Medical Waste Treatment Technologies in Europe.

[7] Ogola J.S., Chimuka L., Tshivhase S., Kumar S. (2011). Management of municipal solid wastes: A case study in Limpopo Province, South Africa. Integrated Waste Management.

[8] Department of Environmental Affairs and Tourism (2008). Program for the Implementation of National Waste Management Strategy. Starter Documents for Health Care Waste.

[9] Maseko Q. (2014). Critical Evaluation of Medical Waste Management Policies, Processes and Practices in Selected Rural Hospitals in The Eastern Cape. Master’s Thesis.

[10] Health Professional Council of South Africa (2016). Guidelines for Good Practice in the Healthcare Profession. Guidelines for the Management of Healthcare Waste.

[11] Sharley H. (2006). Osborne Park Hospital Structure Plan: Final Report.

[12] Department of Environment Affairs Health Care Risk Waste Treatment Figures for 2014. http://sawic.environment.gov.za/documents/4946.pdf.

[13] Department of Environmental Affairs (2018). South Africa State of Waste Report. First Draft Report. http://sawic.environment.gov.za/documents/8641.pdf.

[14] Caniato M., Tudor T., Vacari M. (2016). Assessment of health-care waste management in a humanitarian crisis: A case study of the Gaza Strip. Waste Manag..

[15] Hossain M.S., Santhanam A., Nik Norulaini N.A., Omar A.K. (2011). Clinical solid waste management practices and its impact on human health and environment: A review. Waste Manag..

[16] Mahasa P.S., Ruhiiga T.M. (2014). Medical waste management practices in north eastern Free State, South Africa. Hum. Ecol..

[17] Olaifa A., Govender R.D., Ross A.J. (2018). Knowledge, attitudes and practices of healthcare workers about healthcare waste management at a hospital in KwaZulu-Natal. S. Afr. Fam. Pract..

[18] Makhura R.R., Matlala S.F., Kekana M.P. (2016). Medical waste disposal at a hospital in Mpumalanga Province, South Africa: Implications for training of healthcare professionals. S. Afr. Med. J..

[19] Mashao M.S. (2013). Knowledge and Practices of Health Care Workers on Medical Waste Management Disposal at George Masebe Hospital, Waterberg District, Limpopo Province, South Africa. Master’s Thesis.

[20] Limpopo Department of Health and Social Development (2015). Promotion of Access to Information Act (PAIA).

[21] Nemathaga F., Maringa S., Chimuka L. (2008). Hospital solid waste management practices in Limpopo Province, South Africa: A case study of two hospitals. Waste Manag..

[22] Raphela S.F. (2014). Treatment and disposal of medical waste in rural and urban clinics within Polokwane municipality of South Africa. J. N. Gener. Sci..

[23] Vhembe District Municipality, Department of Health and Social Development (2017). Vhembe District Municipality Records.

[24] Reenen B. (2014). IUSS Health Facility Guides: Infrastructure Design for Waste Management in Healthcare Facilities (Abridged).

[25] Limpopo Provincial Government, Department of Health and Social Development Health Care Risk Waste Guidelines.

[26] Jewaskiewitz S. (2013). A Challenging Context. Infrastructure News and Service Delivery. http://www.infrastructurene.ws/2013/05/20/a-challenging-context/.

[27] Ramokate T., Basu D. (2009). Health care waste management at an academic hospital: Knowledge and practices of doctors and nurses. S. Afr. Med. J..

[28] Kuchibanda K., Mayo A.W. (2015). Public health risks from mismanagement of healthcare wastes in Shiyanga Municipality health facilities, Tanzania. Sci. World J..

[29] Kumar R., Somrongthong R., Ahmed J. (2016). Effect of medical waste management trainings on behavior change among doctors versus Nurses and paramedical staff in Pakistan. J. Ayub Med. Coll. Abbottabad.

[30] Rudraswamy S. (2010). Hospital Waste Management among the Staff of Dental Hospitals. Masters’ Thesis.

[31] Otto K., Clements J. (2008). Survey of Generation Rates, Treatment Capacities and Minimal Costs of Healthcare Waste in the 9 Provinces of RSA.

[32] Eastern Cape Development Corporation (2017). Feasibility Plan for a Hazardous Waste Treatment Facility in the Eastern Region of the Easter Cape.

[33] Zimmermann K. (2018). Microwave technologies: An emerging tool for inactivation of biohazardous material in developing countries. Recycling.

[34] Voudrias E.A. (2016). Technology selection for infectious medical waste treatment using the analytic hierarchy process. J. Air Waste Manag. Assoc..

[35] South African Government (1996). The Constitution of the Republic of South Africa.

[36] Sherman A. Waste Management in Public Hospitals. Department of Health, Province of Kwazulu Natal 2016. https://docplayer.net/14395490-Health-care-waste-categories.html.

